# Orthopedic Management of a Class III Malocclusion With Deep Anterior Crossbite and Transverse Discrepancy: A Case Report

**DOI:** 10.1155/crid/4084487

**Published:** 2025-10-10

**Authors:** César Sebastián Arcos-López, Marco González, Byron Villarreal-Ortega, Juan Marcos Parise-Vasco

**Affiliations:** ^1^Postgraduate Programme in Orthodontics, Faculty of Health Sciences Postgraduate Studies, Universidad UTE, Quito, Ecuador; ^2^Faculty of Dentistry, Universidad UTE, Quito, Ecuador; ^3^Public Health and Epidemiology Research Centre (CISPEC), Eugenio Espejo Faculty of Health Sciences, Universidad UTE, Quito, Ecuador

**Keywords:** angle Class III, case report, functional orthopedics, malocclusion, MARPE, mini-implants

## Abstract

Class III malocclusion is a dentoskeletal condition characterized by altered relationships between bones, teeth, and muscular structures. Sagittal and transversal maxillary deficiency is one of the most common patterns, negatively affecting facial esthetics and the psychosocial well-being of the patient. This case report describes an 11-year-old male patient with Class III skeletal malocclusion, a deep anterior crossbite, and a transverse discrepancy. Orthodontic treatment was performed with a mini-implant assisted rapid palatal expansion (MARPE 2S), anchored in the maxilla with two mini-implants, combined with four additional components: two for a face mask and two for the Ertty Gap III protocol, as well as a lip bumper with flanges for 5/16 heavy intermaxillary elastics in Class III mechanics for 24 h a day, attached to a soldered lingual arch. The combination of these orthopedic devices synergistically improved expansion and anteroposterior changes, resulting in significant functional, esthetic, and psychological improvements for the patient.

## 1. Introduction

Class III malocclusion constitutes one of the most challenging orthodontic anomalies, characterized by a mesial relationship of the mandibular dental arch with the maxillary arch, clinically manifesting when the mesiobuccal cusp of the first maxillary molar occludes distal to the mesiobuccal groove of the first mandibular molar [1]. This condition presents with negative overjet in the anterior segment, frequently accompanied by dentoalveolar compensations that include lingual inclination of the mandibular incisors as an adaptive mechanism to the skeletal discrepancy [2].

From a skeletal perspective, Class III malocclusion can manifest as maxillary hypoplasia, mandibular prognathism, or a combination of both factors [3]. The condition typically presents during early childhood as an edge-to-edge incisal relationship or anterior crossbite, with progression often observed during growth spurts [4]. Epidemiological studies indicate that between 42% and 63% of Class III cases involve maxillary retrusion either isolated or combined with mandibular protrusion, significantly impacting facial esthetics and psychosocial well-being [5, 6]. The prevalence of associated transverse discrepancies exceeds 8%–10% in adolescent and adult populations, with posterior crossbite representing the most frequent malocclusion at 13.3% in mixed dentition patients [5].

The diagnostic approach requires a comprehensive clinical and radiographic evaluation to differentiate the skeletal components from the dental components and determine the primary etiological factor. Cephalometric analysis, in conjunction with clinical evaluation, helps identify whether malocclusion originates from maxillary retrusion, mandibular prognathism, or a combination of both factors [7]. Early intervention during the growth period has been advocated to harness growth modification potential and minimize the need for orthognathic surgery in adulthood.

Mini-implant assisted rapid palatal expansion (MARPE) has emerged as an effective modality for addressing transverse maxillary deficiency while minimizing unwanted dental side effects. The MARPE 2S device incorporates anteroposterior extensions for mini-implant adaptation and lateral bars for band soldering, providing skeletal anchorage through two mini-implants positioned in the palatal vault [8]. This design enables greater skeletal expansion with reduced posterior teeth buccal tipping compared to conventional rapid palatal expansion (RPE) appliances.

The Ertty Gap III protocol, developed by Ertty Silva in 2017, represents an innovative intraoral approach specifically designed for Class III skeletal malocclusions in patients aged 10–13 years [9]. This protocol eliminates the need for extraoral face mask appliances or additional skeletal anchoring devices in the mandible, potentially improving patient compliance and acceptance of treatment. The system employs a modified inverted Hyrax expander with an acrylic base positioned over the premaxilla to stimulate anterior projection. In the mandibular arch, the protocol utilizes a lip bumper connected to a lingual arch through Class III intermaxillary elastics, creating a comprehensive force system that directs maxillary growth anteriorly while modulating mandibular development. The treatment protocol involves weekly activations of the maxillary appliance combined with continuous elastic wear throughout the day for 9–11 months, followed by nocturnal use as retention [9].

The biomechanical principles underlying the Ertty Gap III protocol rely on the absence of condylar corticalization at the beginning of treatment, which indicates a high bone metabolism and an optimal response to elastic mechanics [10]. Continuous force application through the intraoral system provides more consistent orthopedic effects compared to intermittent forces typically associated with removable face mask therapy. Furthermore, the protocol addresses both maxillary advancement and mandibular growth modulation simultaneously, potentially offering a more comprehensive correction of the Class III skeletal pattern.

Although both the MARPE and Ertty Gap III protocols are effective individually, there is little literature documenting their combined application. The purpose of this article is to report the case of a patient with a Class III malocclusion presenting with transversal and sagittal maxillary skeletal discrepancies who was treated with a MARPE 2S appliance combined with the Ertty Gap III protocol.

## 2. Case Report

An 11-year-old male patient presented to the Dental Specialities Service of UTE University in Quito, Ecuador, accompanied by his mother. The main reason for the consultation, as reported by his mother, was concern about inadequate maxillary development and a noticeable concave facial profile. The initial medical history did not reveal any relevant personal or family medical history that could influence the diagnosis or treatment plan. Clinically, the patient presented with a deep anterior crossbite associated with maxillary hypoplasia characterized by a bilateral posterior crossbite.

Extraoral clinical examination revealed distinctive facial characteristics consistent with a severe Class III skeletal pattern. The patient presented a severe brachyfacial pattern, quantified by a VERT index of 1.6, characterized by reduced lower facial height and increased masseter muscle development. The facial profile showed marked concavity with evident retrusion of the upper lip and prominence of the chin region. The nasolabial angle appeared obtuse, and the upper lip showed inadequate support with reduced vermillion display at rest.

Intraoral examination revealed bilateral Class III molar and canine relationships according to angle's classification. The patient presented a deep anterior crossbite that affected all maxillary incisors. Initial measurements documented a negative overjet of −2.1 mm and an overbite of −4.7 mm, indicating horizontal and vertical discrepancies. A bilateral posterior crossbite was observed that extends from the canine to the second deciduous molar regions, suggesting a significant transverse maxillary deficiency. The mandibular dental midline coincided with the facial midline, while the maxillary midline showed no significant deviation.

Comprehensive cephalometric analysis was performed using standardized lateral cephalograms taken in the natural position of the head. The analysis revealed significant skeletal discrepancies that confirmed the clinical diagnosis. The SNA angle measured 82°, indicating a normal maxillary position relative to the anterior cranial base. However, the SNB angle of 85° demonstrated relative mandibular prognathism, resulting in an ANB angle of −3°, confirming the Class III skeletal relationship. Maxillary depth was measured at 89° (normal: 90°), while facial depth was recorded at 93° (normal: 87°), further supporting the diagnosis of relative mandibular prognathism with mild maxillary retrusion (Table 1).

The Wits appraisal provided additional confirmation of the skeletal discrepancy, measuring −11.7 mm (normal: −1 ± 1 *mm*), indicating significant maxillomandibular disharmony. Dental measurements revealed compensatory positioning, with the maxillary incisors inclined to 103° relative to the palatal plane (normal: 110°), suggesting retroclination as a compensatory mechanism. The IMPA measured 79° (normal: 90°), indicating the lingual inclination of the mandibular incisors.

Cone-beam computed tomography evaluation was performed to assess transverse dimensions using Penn analysis. The analysis confirmed the presence of significant transverse discrepancies at the skeletal level, with a 4 mm deficiency in maxillary width compared to mandibular dimensions. This finding corroborated the clinical observation of bilateral posterior crossbite and indicated the need for skeletal expansion as part of the comprehensive treatment approach.

The installation of MARPE 2S was performed under local anesthesia using 2% lidocaine with 1:80,000 epinephrine infiltrated in the palatal region. Two mini-implants (PECLAB, Brazil) measuring 1.8 mm in diameter with 7 mm of threaded length and 4 mm of transmucosal profile were placed in the parasagittal area at the level of the third palatal rugae. The implants were inserted at a 45° angle relative to the palatal plane to optimize bicortical engagement and primary stability.

The MARPE 2S appliance featured a 12-mm expansion screw positioned at the level of the maxillary first premolars. The device incorporated four additional components: two anterior extensions for face mask attachment and two posterior extensions designed for integration with the Ertty Gap III protocol. The appliance was cemented using self-curing glass ionomer cement (Meron, Voco GmbH, Germany) to ensure adequate retention during the expansion phase.

**Figure 1:**
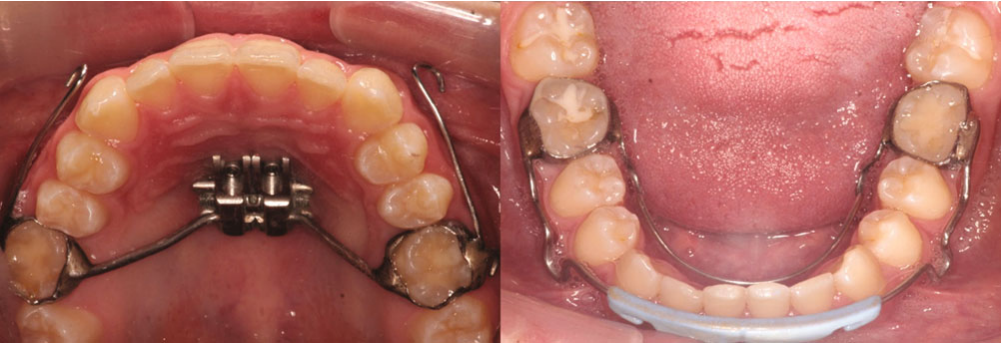
Installation of the MARPE 2S system and mandibular anchorage devices.

In the mandibular arch, the Ertty Gap III component consisted of a lip bumper with lateral flanges specifically designed for intermaxillary elastic attachment. This was complemented by a soldered lingual arch extending from the first permanent mandibular molars. The lingual arch served a dual purpose: to provide anchorage for elastic forces and to prevent potential distalization and distal tipping of the mandibular molars resulting from Class III elastic mechanics (Figure 1).

Following a 1-week healing period to ensure initial osseointegration of the mini-implants, the activation protocol was initiated. The MARPE 2S activation consisted of one quarter turn (0.25 mm) per day for 15 consecutive days, achieving a total expansion of 3.7 at the MARPE screw level. Parents were instructed on the activation procedure and provided with a compliance calendar to ensure adherence to the protocol.

During the expansion phase, Class III intermaxillary elastics (5/16 inch thick, delivering 226 g of force) were initiated. The elastics were worn continuously for 24 h daily, with removal allowed only during school hours to minimize social impact. The force vector was directed from the extensions of the maxillary appliance to the flanges of the mandibular lip bumper, creating an anterior and inferior force on the maxilla while providing a posterior and superior force on the mandible.

A supplementary face mask was prescribed for nocturnal wear to increase the orthopedic effect during the initial 6 months of treatment. The face mask provided an additional protraction force of approximately 400 g per side, directed at a 30° angle inferior to the occlusal plane.

Throughout the treatment period, several minor complications were encountered and conservatively managed. The patient experienced transient pain and localized inflammation around the mini-implant sites during the first 2 weeks after installation. Mucosal ulcerations developed adjacent to appliance components, particularly in the mandibular vestibule region.

These complications were managed by topical application of an analgesic and antiseptic gel (Bucagel, Lamosan, Ecuador) containing 3.0 g of benzocaine for pain relief, 1.0 g of chlorhexidine gluconate (equivalent to 0.2 g of chlorhexidine) for antimicrobial effect, and 0.38 g of zinc lactate for tissue healing promotion. The gel was applied three times daily for 2 weeks until the symptoms were resolved.

Monthly monitoring appointments were scheduled to evaluate the stability of the skeletal anchorage, the periodontal health of the tissues adjacent to the mini-implants, and the progression of expansion. Oral hygiene instructions were reinforced at each visit, with particular emphasis on irrigation around the mini-implant sites using 0.12% chlorhexidine rinse.

**Figure 2:**
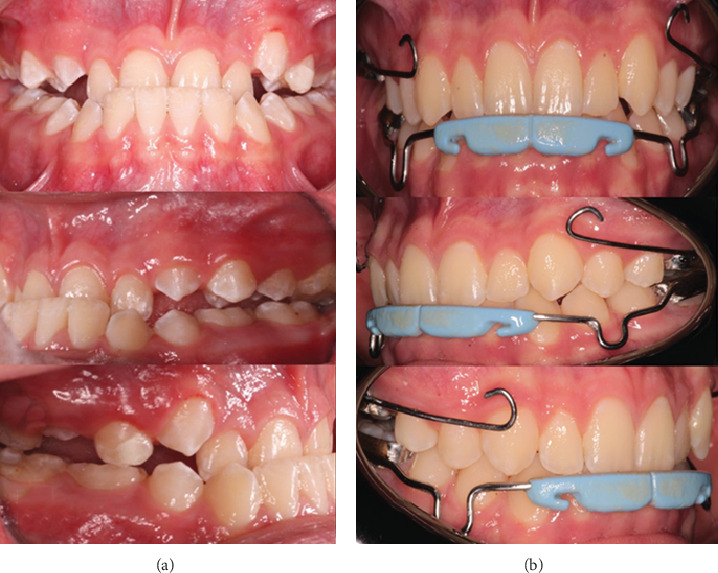
Intraoral photographic sequence of orthopedic treatment. (a) Intraoral images before treatment. (b) Intraoral images during treatment.

Systematic follow-up was conducted throughout the 18-month active treatment period. Monthly clinical evaluations assessed appliance integrity, mini-implant stability, soft tissue health, and patient compliance with elastic wear (Figure 2). Standardized intraoral photographs were obtained at 3-month intervals to document the progress of treatment. Comprehensive radiographic evaluations were performed at 6-month intervals, including lateral cephalograms and panoramic radiographs. These assessments monitored skeletal changes, dental movements, and root integrity. The mini-implants remained stable throughout treatment with no signs of mobility or peri-implant radiolucency.

**Figure 3:**
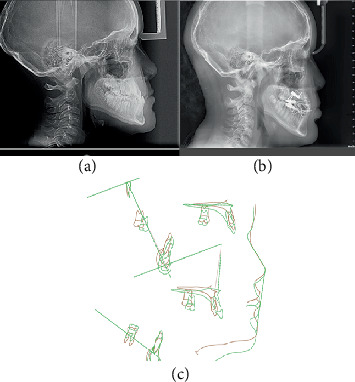
Comparative radiographic evaluation. (a) Radiograph before treatment. (b) Radiograph during treatment. (c) Superimposition of cephalometric tracings.

The posttreatment cephalometric analysis demonstrated significant improvements in both skeletal and dentoalveolar parameters, as detailed in Table 1. Skeletal measurements revealed a successful correction of the Class III relationship. The SNA angle increased by 4°, indicating significant maxillary advancement. The SNB angle showed a slight decrease of 1°, suggesting favorable modulation of mandibular growth. The combined effect resulted in normalization of the ANB angle from -3° to 2°, achieving a Class I skeletal relationship (Figure 3).

The Wits appraisal demonstrated remarkable improvement from −11.7 to −1.8 mm, falling within the normal range and confirming the establishment of harmonious maxillomandibular relationships. Maxillary depth increased favorably from 89° to 93°, exceeding the normative value and indicating effective anterior maxillary positioning.

The dentoalveolar compensation showed favorable resolution. The inclination of the maxillary incisor relative to the palatal plane was normalized from 103° to 110°, eliminating compensatory retroclination. The IMPA increased from 79° to 88°, approaching the ideal value and indicating decompensation of the mandibular incisors.

Clinical examination at the end of the treatment revealed resolution of the anterior and posterior crossbites. The patient achieved bilateral Class I molar and canine relationships with a positive overjet of 2 mm and an overbite of 2 mm. The facial profile showed a marked improvement with improved upper lip support and reduced chin prominence. The nasolabial angle normalized, and the patient demonstrated improved lip competence at rest.

Functional evaluation revealed improved masticatory efficiency with bilateral posterior occlusal contacts. Symptoms of the temporomandibular joint were not reported throughout treatment or at the end of the treatment. Speech articulation, which initially showed mild distortion due to the anterior crossbite, normalized after correction.

**Figure 4:**
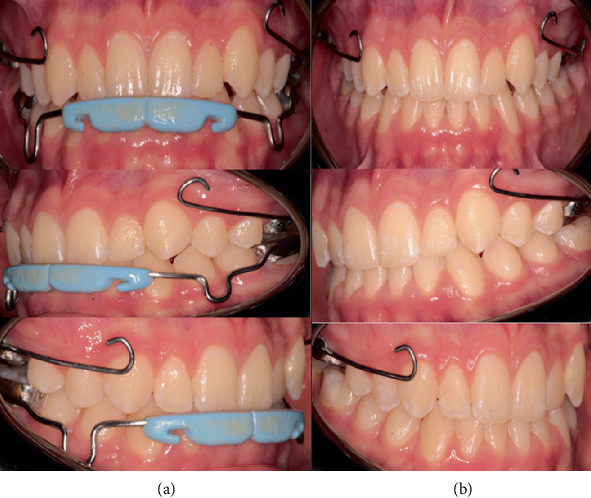
Removal of mandibular appliances. (a) Images before the removal of the mandibular appliance. (b) Images after the removal of the mandibular appliance.

Following 18 months of active treatment, the mandibular appliances were removed, while the maxillary MARPE 2S was maintained in a passive state for an additional 6 months as part of the retention protocol. The patient was scheduled for periodic follow-up evaluations every 3 months during the retention phase to monitor stability and continued growth. Long-term retention planning included the fabrication of a removable maxillary retainer after the removal of MARPE and continued monitoring until growth completion (Figure 4).

**Figure 5:**
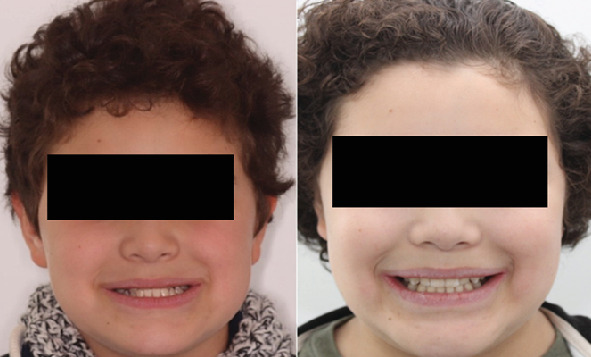
Comparison of frontal photos before and after treatment.

The patient and his legal representative expressed their satisfaction with the results of the treatment, highlighting the favorable changes at both the facial and intraoral levels (Figure 5), noting that the effect was beneficial not only in functional aspects but also in the psychosocial well-being of the patient.

## 3. Ethical Considerations

This case report was conducted after the patient's legal guardian signed the informed consent form and agreed to the treatment plan. Additionally, the guardian agreed to use the patient's clinical information and photographs, which will be presented anonymously for publication in a scientific journal.

## 4. Discussion

This report presents the case of a Class III patient treated with MARPE 2S and the Ertty Gap III protocol, the combination of which resulted in improvements in clinical parameters such as overjet, overbite, molar, and canine class. Imaging demonstrated correction for transverse and sagittal maxillary discrepancies, while psychosocial benefits were evident through increased self-esteem. Early intervention proved essential to use the patient's growth potential, allowing the skeletal discrepancy to be corrected by activating the maxillary suture and modifying the maxillomandibular relationship.

In this case, the combination of both devices produced a more significant effect. Maxillary expansion was achieved with devices anchored to mini-implants in the maxilla and dental anchoring in the mandible, resulting in a maxillary discrepancy and sagittal repositioning [9]. Rapid maxillary expansion (RME) is generally the standard treatment for patients with transverse maxillary deficiency. RME can be performed successfully in young patients without closed midpalatal sutures [11].

A systematic review of the literature found that patients who underwent conventional RPE had a greater loss of buccal alveolar bone thickness (BT) than those treated with MARPE. The subgroup analyses showed significant differences in both premolar regions, right and left, but no significant differences in the molar regions [12]. Treatments with MARPE 2S in combination with a face mask have shown positive results, supporting its appropriate use. In the literature, anterior crossbites have improved spontaneously after MARPE. The main effects are anterior displacement of the maxilla and posterior rotation of the mandible [11]. MARPE not only corrects transverse deficiencies but also induces changes in anteroposterior and vertical dimensions. Posterior mandibular rotation results in a slight increase in total anterior facial height [13]. This approach can reduce the severity of borderline cases and expand the scope of orthodontic treatment for Class III malocclusions.

Although effective, MARPE is not without adverse effects and complications. Reported adverse effects include epistaxis, inflammation and swelling of the palatal mucosa, difficulty cleaning, soft tissue impingement, microimplant loosening, tinnitus, expander distortion, suture failure, and asymmetric expansion [11, 14]. In this case, minor adverse effects such as pain, inflammation, and ulceration were observed.

In the present case, a lingual arch combined with a labial bumper was used as an alternative to mandibular mini-implants to treat a Class III malocclusion. However, the literature suggests that after MARPE and initial alignment, the use of temporary anchor devices (TADs) on mandibular buccal shelves is effective for molar distalization to alleviate crowding [14]. For the lower arch, the Ertty Gap III protocol was followed using a lingual arch, a lip bumper, and 5/16 heavy elastics worn most of the day. The literature reports excellent results for this protocol in maxillary protraction in children up to 13 years of age. The esthetic results are satisfactory compared to other devices for the same purpose [10]. The intraoral system promotes better patient compliance, with continuous force application producing better results than the intermittent forces typically seen with other devices [9]. However, certain limitations and contraindications must be considered. Disk displacement without reduction is an absolute contraindication, as is advanced skeletal age. The absence of condylar corticalization must be confirmed prior to treatment [9]. This is crucial, as it indicates high bone metabolism, which optimizes the effectiveness of elastic mechanics [10].

The combined effect of transverse expansion and sagittal protraction appeared to facilitate greater maxillary advancement than either modality alone. The initial expansion may have disarticulated the circummaxillary sutures, potentially enhancing the maxillary response to protraction forces. This hypothesis is consistent with previous studies showing that maxillary expansion can facilitate subsequent orthopedic movements [11]. The 4° improvement in SNA and the 9.9 mm improvement in Wits appraisal exceed typical results reported for face mask therapy alone, which generally achieve 2°–3° of change in SNA [13].

An important consideration in this case was the growth potential of the patient. At 11 years of age with active growth remaining, the timing was optimal for orthopedic intervention. The severe brachyfacial pattern suggested a horizontal growth vector, which typically responds favorably to Class III treatment mechanics. The slight posterior rotation of the observed mandible (1° decrease in SNB) contributed to the correction without significantly affecting the vertical dimension, maintaining the favorable brachyfacial characteristics of the patient.

The treatment protocol demonstrated good patient acceptance despite the complexity of the appliance system. The intraoral nature of the Ertty Gap III components eliminated the social stigma associated with the use of a face mask, likely contributing to the excellent compliance observed. The ability to remove elastics during school hours further enhanced acceptability while maintaining therapeutic effectiveness through continuous wear during nonschool hours.

The main limitation of this case was the short follow-up and treatment period. Male patients in active growth require regular evaluation to monitor their growth peaks, with significant skeletal changes likely to occur around 21 years of age. The case remains active, and final outcomes will be determined in the future.

One of the strengths of this case is the presentation of results from a combination of devices whose synergy has not been described in the literature. In this case, the combination of two orthopedic devices improved disjunction effects, anteroposterior changes, and clinical, imaging, and psychological outcomes for the patient. More research is recommended, including randomized clinical trials, to determine the true effectiveness of this intervention. In addition, the biomechanical interaction between transverse expansion and sagittal protraction requires further elucidation. Finite element analysis could provide information on stress distribution patterns and help optimize force vectors for maximum therapeutic effect. Understanding these mechanical principles would enable the refinement of the protocol and potentially reduce the duration of treatment.

## 5. Conclusions

This case report demonstrates the successful three-dimensional correction of a Class III malocclusion using a novel combination of the MARPE 2S and Ertty Gap III protocols. The 18-month treatment resulted in normalized skeletal relationships, resolved dental crossbites, and a significant improvement in facial esthetics. The combined use of simultaneous transverse expansion and sagittal protraction suggests potential advantages over individual treatment modalities. This combined protocol offers a promising alternative for the comprehensive orthopedic management of Class III malocclusion in growing patients. Integrating skeletal anchorage through mini-implants with continuous intraoral force application addresses concerns regarding both efficiency and patient acceptance. Although these initial results are encouraging, further research involving larger sample sizes and longer follow-up periods is essential to establish treatment protocols and predict long-term stability.

## Figures and Tables

**Table 1:** Comparison of cephalometric parameters before and after orthopedic treatment.

**Measurement**	**Pretreatment**	**Posttreatment**	**Normative value**	**Change**
SNA	82°	86°	82°	+4°
SNB	85°	84°	80°	−1°
ANB	−3°	2°	2°	+5°
Maxillary depth	89°	93°	90°	+4°
Facial depth	90°	91°	87°	+1°
Wits appraisal	−11.7 mm	−1.8 mm	−1 ± 1 *mm*	+9.9 mm
Upper incisor to palatal plane	103°	110°	110°	+7°
IMPA	79°	88°	90°	+9°

## Data Availability

Data sharing is not applicable as no new data is generated.
